# Harvesting Friction Energy on Zinc Oxide and Zinc Oxide/Europium Oxide Sol-Gel Catalysts for Tribocatalytic Paracetamol Degradation

**DOI:** 10.3390/molecules30112265

**Published:** 2025-05-22

**Authors:** Dobrina Ivanova, Hristo Kolev, Ralitsa Mladenova, Bozhidar I. Stefanov, Nina Kaneva

**Affiliations:** 1 Laboratory of Nanoparticle Science and Technology, Department of General and Inorganic Chemistry, Faculty of Chemistry and Pharmacy, University of Sofia, 1 James Bourchier Blvd., 1164 Sofia, Bulgaria; dobrina.k.ivanova@gmail.com; 2 Institute of Catalysis, Bulgarian Academy of Sciences, “Acad. G. Bonchev” Str., Bldg. 11, 1113 Sofia, Bulgaria; hgkolev@ic.bas.bg (H.K.); ralitsa@ic.bas.bg (R.M.); 3 Department of Chemistry, Technical University of Sofia, 8 Kliment Ohridsky Blvd., 1756 Sofia, Bulgaria; b.stefanov@tu-sofia.bg

**Keywords:** tribocatalysis, sol-gel ZnO, ZnO/Eu_2_O_3_, paracetamol

## Abstract

In the natural environment, mechanical energy is widely available as a sustainable and green energy source. In this paper, we successfully convert mechanical energy on ZnO and ZnO/Eu_2_O_3_ tribocatalysts via a friction route. Electrons were transferred across the contact interface when the catalyst particles and the polytetrafluoroethylene (PTFE)-sealed magnetic bar rubbed against each other under magnetic stirring. At the same time, holes were left on the catalyst while the PTFE absorbed the electrons. Similar to photocatalysis, organic pollutants can be effectively oxidized by the holes in the valence band of sol-gel catalysts due to their strong oxidative ability. The tribocatalytic tests demonstrated that ZnO and ZnO/Eu_2_O_3_ could eliminate organic analgesics (paracetamol) under magnetic stirring in the dark. By controlling the quantity of rare earth elements (1, 2, and 3 mol%), stirring speed, and the number of magnetic rods, we could further enhance the tribocatalytic performance. In addition to developing a green tribocatalysis approach for the oxidative purification of organic pollutants, this work offers a potential route for converting environmental mechanical energy into chemical energy, which could be used in sustainable energy and environmental remediation.

## 1. Introduction

The increasing demand for clean and sustainable water resources, combined with the persistent contamination of aquatic systems by pharmaceutical residues, has driven the development of various advanced oxidation processes (AOPs) for the degradation of organic pollutants [1,2]. Techniques that utilize physical activation sources—such as light, ultrasound, electric, and magnetic fields—have attracted considerable interest for their efficiency in degrading recalcitrant compounds [3,4,5,6,7]. In particular, the contamination caused by pharmaceuticals such as antibiotics is of growing concern due to their persistence and potential to promote antibiotic resistance. While conventional AOPs, such as photocatalysis and electrochemical oxidation, are effective, they often rely on external energy sources such as light or electricity, limiting their practicality in ambient or off-grid conditions [8,9,10,11,12,13,14].

Recently, mechanical energy-driven processes, including tribocatalysis have emerged as sustainable and light-independent alternatives. Tribocatalysis is a mechanochemical process that utilizes friction-induced charge generation to produce reactive oxygen species capable of oxidizing organic pollutants [15,16,17]. Its mechanism shares conceptual similarities with piezocatalysis and mechanochemistry, where mechanical forces are harnessed to drive redox reactions [18,19].

Zinc oxide (ZnO), a wide-bandgap semiconductor with intrinsic piezoelectric properties, is widely studied for such applications due to its low cost, chemical stability, and environmental friendliness [20]. Therefore, we believe that the triboelectric properties of ZnO could be used to oxidize pollutants, achieving the frictional conversion of mechanical energy to chemical energy [21,22,23,24]. Thus, the use of nanomaterials’ frictional effects to degrade pollutants has emerged as a novel concept. ZnO, an excellent semiconductor material with great chemical stability and environmental friendliness, can significantly affect catalytic activity due to its various morphologies [21,25]. In fact, more active sites are found in nanomaterials with a higher specific surface area, and these sites are crucial for accelerating the breakdown of organic compounds. The tribocatalytic efficiency of zinc oxide can be significantly enhanced through doping with rare-earth elements, which can improve charge carrier separation, tailor surface states, and modulate the band structure. Europium (Eu), capable of existing in multiple oxidation states (Eu^3+^ and Eu^2+^), is a promising dopant that can influence the surface chemistry and electronic properties of ZnO-based materials [26]. While Eu-doped ZnO has shown promising activity in photocatalytic and luminescent applications, its behavior under tribocatalytic conditions remains largely unexplored. Furthermore, there is a lack of systematic studies on how tribocatalysis affects the surface chemistry of doped ZnO systems, including changes in oxidation state, surface composition, and defect generation.

In this study, we investigate the tribocatalytic degradation of paracetamol using pure and Eu^3+^-modified ZnO nanomaterials (0, 1, 2, and 3 mol%) synthesized by a sol-gel method. The materials are evaluated under dark stirring conditions to simulate ambient mechanical activation. The impact of Eu doping on the catalytic activity is correlated with changes in surface chemistry and structural properties. A comprehensive suite of characterization techniques—X-ray photoelectron spectroscopy (XPS), electron paramagnetic resonance (EPR), scanning and transmission electron microscopy (SEM and TEM), X-ray diffraction (XRD), energy-dispersive X-ray spectroscopy (EDS), and UV–vis spectroscopy—is employed to elucidate the relationship between material structure and tribocatalytic performance. This work offers new insights into the role of rare-earth elements in enhancing tribocatalysis and demonstrates the potential of Eu-doped ZnO as an effective catalyst for the degradation of pharmaceutical pollutants in water.

## 2. Results and Discussion

### 2.1. Structure Analysis

**Figure 1:**
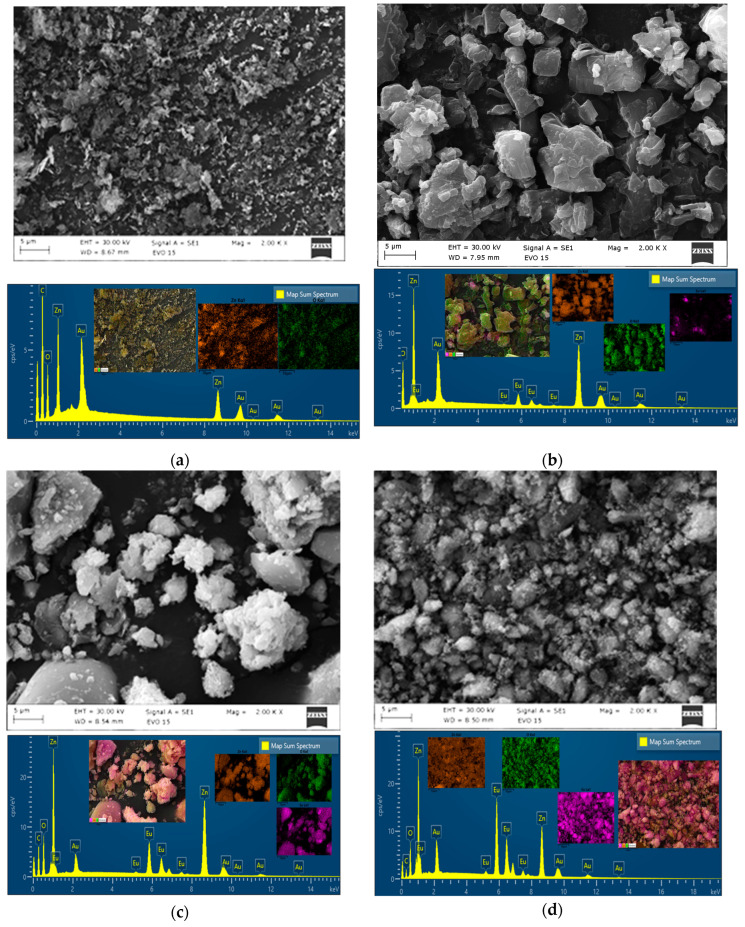
SEM micrographs and EDS spectra of ZnO (**a**), ZnO/1 mol% Eu_2_O_3_ (**b**), ZnO/2 mol% Eu_2_O_3_ (**c**), and ZnO/3 mol% Eu_2_O_3_ (**d**). The insets show composition maps of Zn, O, and Eu.

The morphology of pristine and Eu_2_O_3_-modified ZnO particles is investigated using scanning electron microscopy (SEM), as shown in Figure 1. Identifying the structural morphological changes between pure ZnO and ZnO modified with europium is straightforward. A generally homogeneous dispersion of particles with varying sizes and shapes is visible in the ZnO/Eu_2_O_3_ SEM images. The europium ions strongly promote crystal nucleation. One possible explanation for this trend is the difference in ionic radii between europium and zinc [27]. The results of Chao et al. [28] are similar to the SEM results obtained.

Energy-dispersive X-ray spectroscopy (EDS) analysis is carried out on the ZnO/Eu_2_O_3_ samples to verify the presence of Eu on the surface of the ZnO. Figure 1 displays representative spectra. The atomic percentages of elements in the four types of tribocatalysts are established: ZnO (Zn 12.18 at%, O 87.82 at%), ZnO/1 mol%, Eu_2_O_3_ (Zn 21.07 at%, O 77.22 at%, Eu 1.71 at%), ZnO/2 mol%, Eu_2_O_3_ (Zn 25.40 at%, O 70.90 at%, Eu 3.70 at%), and ZnO/3 mol%, Eu_2_O_3_ (Zn 22.26 at%, O 73.90 at%, Eu 4.48 at%). Eu content rises marginally for particles treated with the highest concentration. The four catalysts were prepared on a carbon patch and coated with gold during their characterization. For this reason, carbon and gold peaks are observed in the EDS spectra. No other elemental peaks were found; the purity of the ZnO and ZnO/Eu_2_O_3_ tribocatalysts was confirmed.

**Figure 2:**
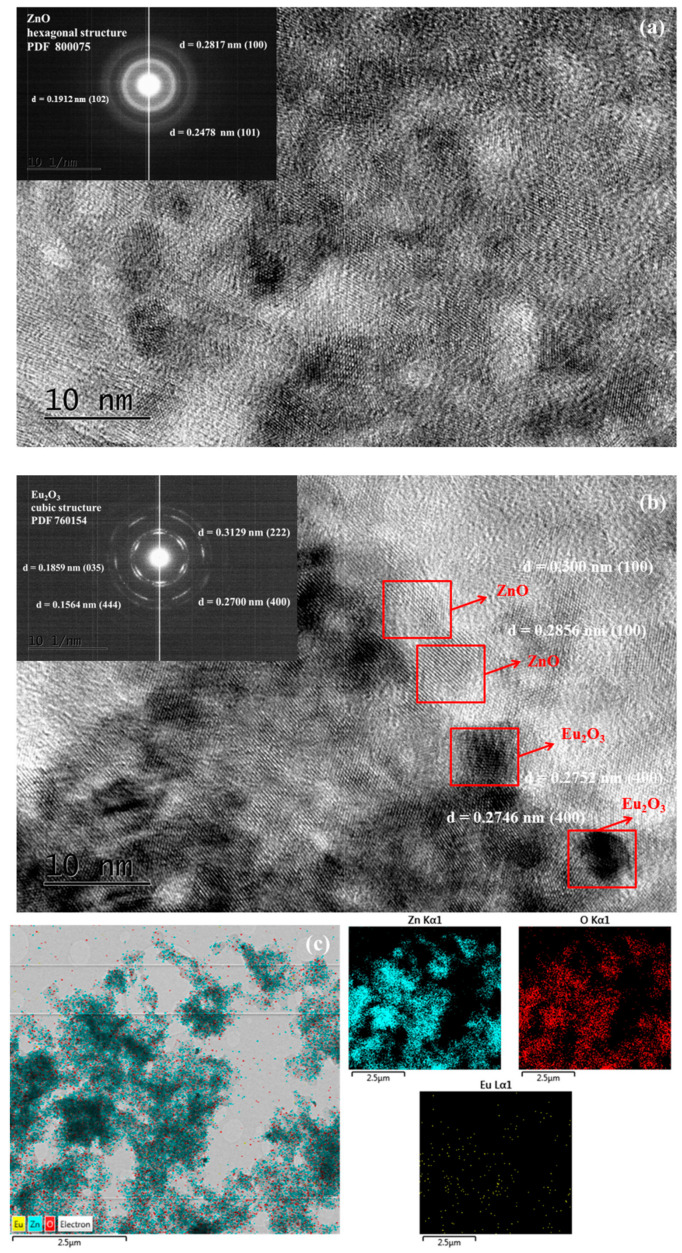
HRTEM image of ZnO and ZnO/Eu_2_O_3_ tribocatalysts. The inset in (**a**,**b**) is the corresponding SAED pattern. (**c**) The composition map of elements using STEM-XEDS analysis.

Figure 2 displays TEM images of the two distinct phases (ZnO and Eu_2_O_3_). The ZnO particles, exhibiting a hexagonal wurtzite structure, are depicted in Figure 2a. The modified ZnO/Eu_2_O_3_ is presented in Figure 2b. The high-resolution TEM (HRTEM) of the two-phase interface, located in the chosen region of Figure 2b, is shown in Figure 2. The (400) plane of Eu_2_O_3_ single crystals, PDF 76-0154 (cubic structure), corresponds to the continuous (400) atomic plane.

The hexagonal wurtzite structure of ZnO is confirmed by selected area electron diffraction patterns (SAED, insets in Figure 2a,b) derived from TEM images, which align with the XRD data. The insets reveal no change in the interplanar distances of the crystal lattice of both pure and modified ZnO catalysts (d100–0.2817 nm, d101–0.2478 nm, and d102–0.1807 nm). The composition map generated by STEM-XEDS analysis was utilized to examine the distribution of elements in the sol-gel sample. The findings from the mapping study indicate that the Zn, O, and Eu elements coexist and are uniformly distributed (Figure 2c). These results also demonstrate that the Eu doping in the ZnO nanoparticles was uniform.

**Figure 3:**
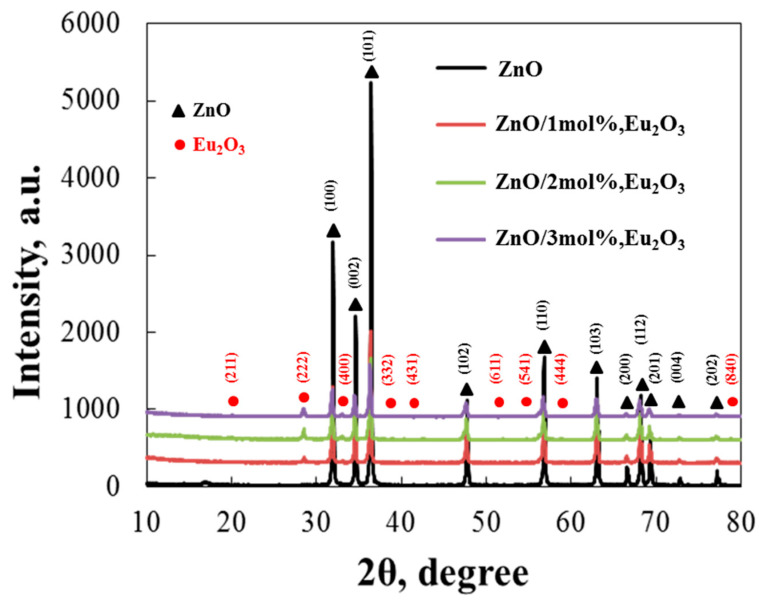
XRD patterns of ZnO/Eu_2_O_3_ samples prepared by the sol-gel method at europium concentrations of 0, 1, 2 and 3 mol%.

The XRD patterns of the prepared ZnO and ZnO/Eu_2_O_3_ samples are shown in Figure 3. Diffraction from the (100), (002), and (101) planes of the hexagonal wurtzite structure of ZnO resulted in three prominent peaks. The indices (102), (110), (103), (200), (112), (201), and (202) also appear in the samples as additional diffraction peaks. This analysis indicates that the wurtzite structure, which possesses a polycrystalline nature, is present in ZnO produced with varying europium concentrations. For pure ZnO nanoparticles, the observed diffraction peaks were very similar to the data reported by Wang, R. H., et al. [29] and Aydin et al. [30]. The XRD patterns demonstrated that the (101) plane was the preferred growth orientation. Although only two diffraction peaks, (222) and (400) (cubic Eu_2_O_3_ phase JCPDS Card No. 34-0392 and 76-0704), were detected, other europium oxide peaks were not observed. No peaks from other impurities were found, suggesting that Eu_2_O_3_ NPs are present on the ZnO surface but were not integrated into the ZnO lattice [31]. As shown in Figure 3, the intensities of the diffracted peaks decreased as the Eu concentration increased to 3 mol%, indicating a reduction in the sample’s crystallization. This decrease in crystallinity is likely due to the significant ionic size difference between Zn^2+^ and Eu^3+^ [32,33]. Furthermore, Table 1 presents all of the calculated structural parameters for pure and modified tribocatalysts.

The addition of europium ions had no discernible effect on the crystal size, as shown in the XRD results. Nevertheless, the intensity of the ZnO peaks dropped when the particles were modified. The average crystallite size shrank as the concentration of europium increased, suggesting that the crystalline lattice remained relatively stable. The structural parameters of the pure and 1, 2, and 3 mol% europium-modified sol-gel samples are presented in Table 1. The pure ZnO’s unit cell parameters were found to be extremely similar to those of the europium-modified samples. Tensile strain was represented by positive values in the computations. According to the estimations in Table 1, the modified particles exhibited a higher magnitude tensile strain than the pure samples, and the strain in the sol-gel-derived samples was tensile.

Electron spectroscopy for chemical analysis (ESCA), more commonly known as X-ray photoelectron spectroscopy (XPS), was employed to investigate the surface atomic composition and oxidation states of elements in the ZnO/xEu (x = 0, 1, 2, and 3 mol%) samples before and after tribocatalytic testing. Our analysis focuses on the Eu 3d_5/2_ core level, due to europium’s role in modifying the ZnO surface, and the O 1s core level, where changes were observed with increasing europium content. The Zn 2p core level exhibited no significant variations and is therefore not discussed in detail; its binding energy remains consistent with that reported for pure ZnO, as shown in Ivanova et al. [34]. Survey and high-resolution X-ray photoelectron spectra of Eu 3d and O 1s core levels for ZnO/xEu samples (x = 0, 1, 2, and 3 mol%) before and after tribocatalytic testing are shown in Figure 3. The survey spectra measured using AlKα radiation identify core level peaks corresponding to zinc (Zn2p, Zn3p, Zn3s and Auger peak ZnLMM), oxygen (O 1s, O2s), and europium (Eu3d, Eu4d), which constitute the studied surfaces. Additionally, adventitious carbon (C 1s core level) as a contamination from the vacuum system is also detected. Further insights can be obtained from the high-resolution XP spectra.

**Figure 4:**
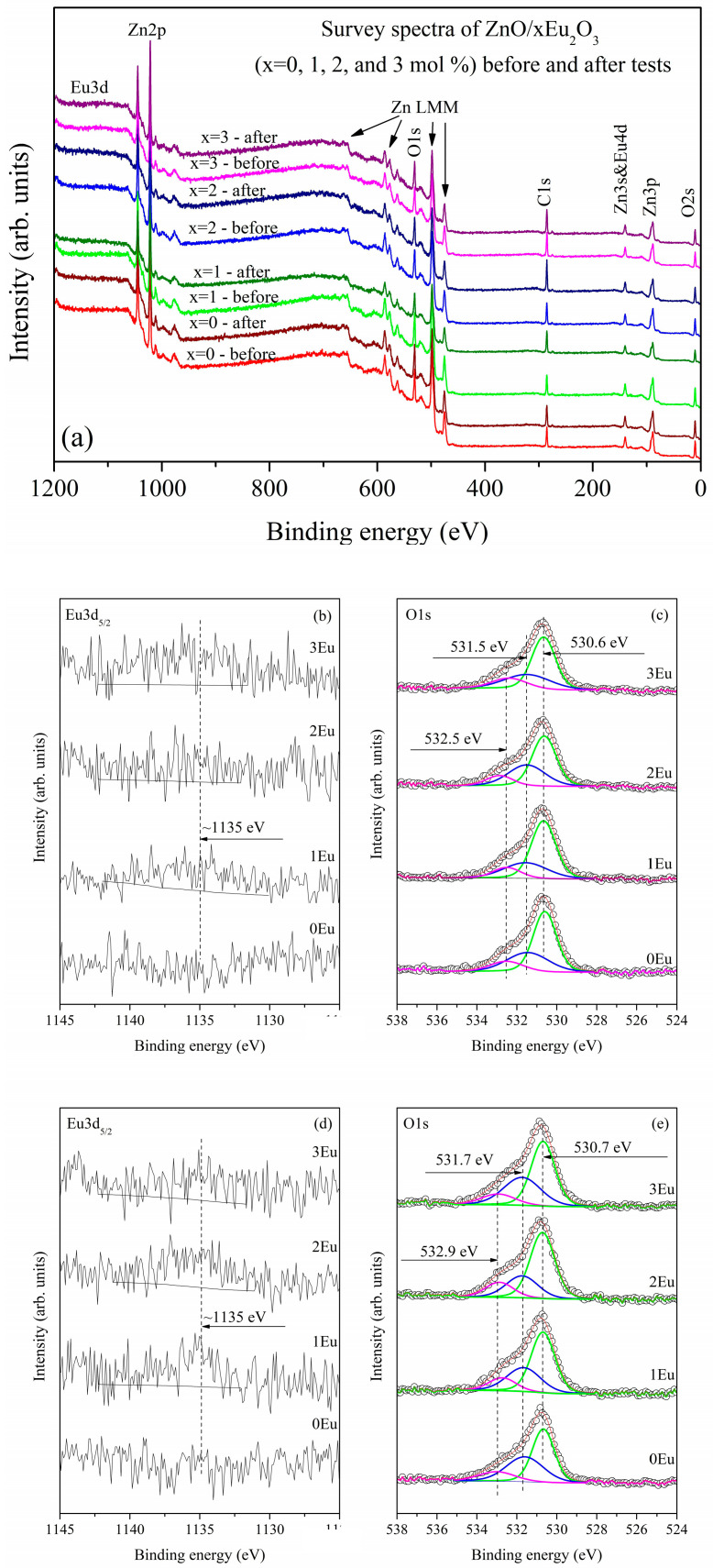
(**a**) Survey and high-resolution X-ray photoelectron spectra of (**b**) Eu 3d and (**c**) O 1s core levels for ZnO/xEu samples (x = 0, 1, 2, and 3 mol%) before and after tribocatalytic testing (**d**) Eu 3d and (**e**) O 1s core levels for ZnO/xEu samples (x = 0, 1, 2, and 3 mol%).

Figure 4 presents high-resolution XPS spectra of (b) Eu 3d_5_/_2_ and (c) the curve-fitted O 1s region for the samples before tribocatalytic testing, with increasing europium concentration from bottom to top. The Eu 3d_5_/_2_ spectra show an intensity increase and the emergence of a peak-like structure centered around 1135 eV, indicating the presence of Eu^3+^ ions in the 1Eu, 2Eu, and 3Eu samples [34]. Additionally, a feature near 1127 eV is attributable to Eu^2+^ species [35], suggesting the coexistence of Eu^2+^ on the sample surfaces. The presence of Eu^2+^ is further confirmed by EPR analysis. Figure 4c shows the curve-fitting of the O 1s core level spectra into three components. The most intense peak (green) is assigned to lattice oxygen in Zn–O bonds (~530.7 eV) [34]. The second peak (blue), located at ~531.5 eV, corresponds to non-lattice oxygen species such as oxygen vacancies and low coordination sites. The third component (magenta), found between 532.5–533.0 eV, is attributed to oxygen from adsorbed water.

Table 2 summarizes the surface atomic concentrations (at.%), binding energies (BE, eV), and associated oxidation states or bonding environments for the ZnO/xEu samples prior to tribocatalytic testing. Despite the relatively poor signal-to-noise ratio in the Eu 3d_5_/_2_ spectra, the surface europium concentration shows a clear increasing trend with nominal doping level. Meanwhile, the concentrations of non-lattice oxygen and adsorbed water remain relatively unchanged within error margins, indicating minimal surface modification at low europium levels. Figure 4d,e presents the Eu 3d_5_/_2_ and O 1s spectra of the samples after tribocatalytic testing, again shown with increasing europium content from bottom to top. The Eu 3d_5_/_2_ spectra retain a prominent peak at ~1135 eV, confirming the presence of Eu^3+^ ions post-reaction. However, no distinct feature is observed near 1127 eV, suggesting the absence of Eu^2+^ species after testing. This observation is consistent with EPR results, which also failed to detect Eu^2+^ after the tribocatalytic process. In Figure 4e, the same color scheme is used for O 1s peak fitting: green for Zn–O lattice bonds (~530.7 eV), blue for non-lattice oxygen (~531.7 eV), and magenta for adsorbed water (~532.9 eV).

Table 3 presents the surface atomic concentrations and corresponding chemical states for the post-reaction samples. Interestingly, the trend of increasing europium concentration observed before testing is no longer present. For instance, the 3 mol% Eu sample shows the lowest measured surface concentration (0.16 at%), indicating a possible redistribution or loss of europium during tribocatalysis. A new correlation is observed between europium content and non-lattice oxygen: samples with lower surface europium exhibit higher concentrations of non-lattice oxygen.

Comparison of the XPS data before and after tribocatalytic testing indicates that Eu^3+^ is the dominant surface species in both cases, while Eu^2+^ is present only prior to testing. Furthermore, a correlation between surface europium and non-lattice oxygen is evident after tribocatalysis—lower europium concentrations are associated with increased non-lattice oxygen levels. This trend was not observed in the pre-reaction samples, where the europium content increased without significantly affecting non-lattice oxygen. These findings suggest that non-lattice oxygen species, including oxygen vacancies and low coordination sites, may play an active role in the tribocatalytic process.

**Figure 5:**
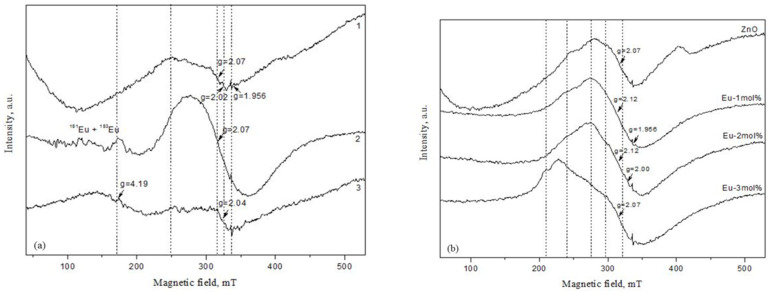
(**a**) EPR spectra of ZnO modified with 1 mol% Eu_2_O_3_ (1), 2 mol% Eu_2_O_3_ (2), and 3 mol% Eu_2_O_3_ (3); (**b**) EPR spectra of undoped ZnO and ZnO modified with 1, 2 and 3 mol% Eu_2_O_3_. The signal gain in the europium samples is one time greater than the undoped sample.

EPR spectroscopy was used to investigate the influence of increasing Eu_2_O_3_ content on the electronic properties of ZnO, as well as on the local site symmetry around doped paramagnetic ions (rare earth ions). Figure 5a shows the X-band EPR spectra of ZnO doped with increasing amounts of Eu_2_O_3_ quantity (1, 2, and 3 mol%), recorded at room temperature. The Eu^2+^ ion, with an electron configuration of 4f7 and a ground state of 8S = 7/2, is an EPR active species. Naturally occurring europium consists of two isotopes: 151Eu (48% abundance) and 153Eu (52% abundance), with a nuclear spin I = 5/2. This led to the splitting of the absorption line into 2I + 1 hyperfine components due to an electron-nucleus Zeeman interaction. Several resonance absorptions may occur depending on the strength of the crystal field [36]. A crystal field stronger than the Zeeman interaction typically leads to resonance absorptions at g > 2, whereas a weak crystal field results in signals near g ≈ 2 [37]. In the current EPR study, Eu^2+^ signals at g > 2 were observed in the ZnO samples doped with 2 and 3 mol% Eu_2_O_3_, suggesting that some Eu^2+^ ions are located at lattice sites with strong crystal fields, likely with octahedral symmetry. Eu^2+^ tends to substitute Zn^2+^ ions at octahedral sites within the host lattice [38]. Several lines with resolved hyperfine structure were recorded at the range of magnetic fields from 80 mT to 180 mT for the ZnO/Eu_2_O_3_ (2 mol%), which can be attributed to transitions of Eu^2+^. The observed spectrum, consisting of around 12 overlapping lines, arises from the superposition of signals of Eu with natural isotopic composition. A similar spectrum was reported for Eu:YAG single crystals by Petrosyan et al. [39]. In the ZnO/Eu_2_O_3_ (3 mol%) sample, a broad unresolved signal was observed at g ≈ 4.2. A similar signal was previously reported by Reddy et al. in ZnO:Eu (0.1 mol%) nanopowders [40]. It is reasonable to suggest that an increased Eu_2_O_3_ content in the samples leads to a higher amount of Eu^2+^ ions, which in turn results in increased intensity of the EPR peak at g ≈ 4.2. Trivalent Europium (4f^6^, ^7^F_0_) does not give an EPR signal since it is diamagnetic in nature.

A broad, asymmetric signal was observed in all samples, most likely due to the overlap of several signals with close g-factors. This complicates the precise identification of all recorded signals. A resonance at g = 2.07 (observed in spectra 1 and 2 in Figure 5a) may originate from O^2−^ species formed due to traces of oxygen being present in the system [41] or from OH^•^ radicals adsorbed on the catalyst surface [42]. Additionally, a weak signal at g = 2.02 was recorded in spectrum 1, possibly associated with oxygen radicals, which typically exhibit g-values between 2.00 and 2.03 [42]. While in the ZnO/Eu_2_O_3_ (3 mol%) sample, a weak resonance line at 325 mT (g = 2.04) was observed (spectrum 3). A similar signal was reported in N-implanted ZnO films and attributed to V_0_–V_Zn_ divacancy [43]. Stan et al. detected a signal at g = 2.03, which was attributed to coupled defects, such as negatively charged zinc vacancy-interstitial zinc (V-_Zn_:Z_ni_^0^) complexes. A weak, narrow singlet at g = 1.956 was detected in all samples and can be attributed to shallow effective mass donor (SD) centers in ZnO. Some studies have assigned the resonance at g = 1.96 to bulk defects originating from negatively charged Zn vacancies [44] or to oxygen vacancy-related defects in ZnO. An increase in europium content led to a slight increase in the intensity of the signal with g_eff_ = 1.956, with similar intensities recorded for the 2 and 3 mol% doped samples.

Figure 5b presents the EPR spectra of undoped and Eu_2_O_3_-doped ZnO samples after tribocatalytic treatment. Some differences are observed between the EPR spectra recorded before and after mechanical activation. The EPR measurements show the absence of resonance signals attributable to paramagnetic europium species, suggesting that Eu^2+^ ions are either not present in detectable concentrations or have been oxidized to the diamagnetic Eu^3+^ state. This assumption is supported by XPS analysis, which indicates a shift in the Eu^3+^ ↔ Eu^2+^ redox equilibrium toward the trivalent state. Similar to the pre-catalysis spectra, overlapping signals are observed, complicating detailed analysis via X-band EPR. Therefore, only distinct EPR signals are discussed. A signal at g = 2.07 appears in both undoped ZnO and the 3 mol% Eu_2_O_3_-doped sample, while a different signal with g = 2.12 is observed in ZnO doped with 1 and 2 mol% Eu_2_O_3_. The g = 2.07 signal was previously discussed and is attributed to O^2−^ species or OH^•^ radicals adsorbed on the catalyst surface. The shift from g = 2.145 to 2.12 is likely due to overlapping signals and is commonly associated with Zn vacancies [40]. Singly ionized oxygen vacancies (g ≈ 2.000) are most likely present in all samples [43,45]. As in the pre-catalysis spectra, shallow donor (SD) centers are observed at g = 1.956. Their concentration increases in the 2 and 3 mol% Eu-doped ZnO samples, with the 3 mol% sample exhibiting approximately twice the intensity compared to the undoped and 1 mol% doped samples. As mentioned, the EPR signal with g_eff_ = 1.956 is responsible for the donor states. Since the intensity of this signal depends on the europium concentration, it can be assumed that Eu atoms are involved in the structure of these donor centers. The remaining unidentified signals are most likely due to ZnO impurities containing metal ions.

**Figure 6:**
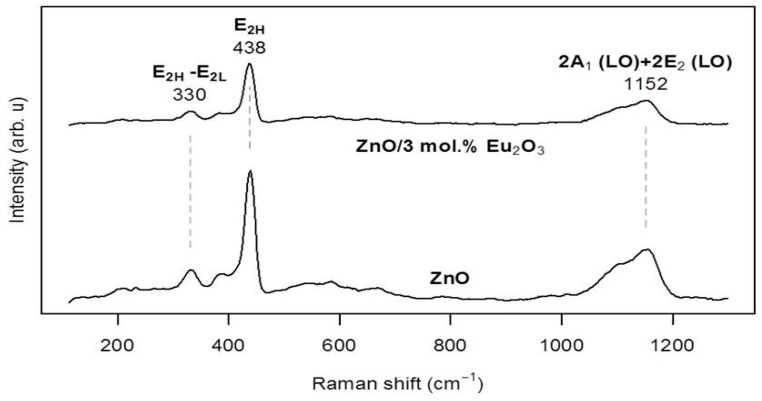
Raman spectra of ZnO and ZnO/3 mol%, Eu_2_O_3_ samples.

Figure 6 presents a comparison of the Raman spectra of the pure ZnO powder and the ZnO/Eu_2_O_3_ sample modified with 3 mol% Eu_2_O_3_, recorded in the 110–1300 cm^−1^ range. The Raman data confirm the hexagonal wurtzite phase composition of ZnO, consistent with the XRD patterns. The most prominent Raman peaks appear at 330 and 438 cm^−1^, corresponding to the E2H–E2L and E2H Raman modes, respectively [46], followed by a broad feature at 1152 cm^−1^, attributed to the 2A1(LO) and 2E2(LO) modes. No significant spectral changes are observed in the Eu_2_O_3_-doped powder, regardless of the dopant concentration, except for a reduction in intensity and broadening of the Raman modes. This observation is consistent with the XRD analysis. A slight redshift (~1 cm^−1^, discernible but below the resolution of the Raman instrument used) of the observed Raman modes is also noted, which may indicate partial substitution of lattice Zn atoms by Eu [47].

### 2.2. Optical Analysis

**Figure 7:**
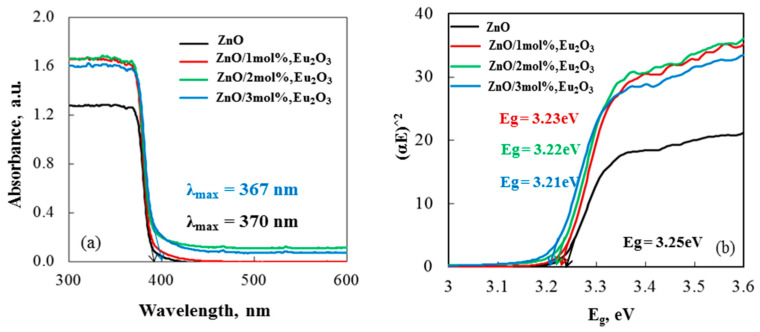
(**a**) UV–visible patterns of tribocatalysts and (**b**) linear plots of (αhv)^2^ versus E_g_ for tribocatalysts.

UV–vis absorption spectroscopy was employed to examine the optical characteristics of the tribocatalysts. Figure 7a indicates that pure ZnO exhibited an absorption band in the UV region at approximately 370 nm. The interaction between Eu and the semiconductor resulted in a slight shift of the absorption band (λ_max_ = 367 nm) in the europium-modified samples. This interaction was attributed to strong interfacial electronic coupling between ZnO and Eu_2_O_3_. Consequently, the loss of light energy was further minimized [48].

The three samples exhibited band gap energies of 3.23, 3.22, and 3.21 eV for 1, 2, and 3 mol% Eu-modified ZnO, respectively, according to the relationship E_g_ (eV) = 1240/λmax (nm), Figure 7b [49]. The value of the band gap energies decreased from 3.25 eV for pure ZnO to 3.21 eV for ZnO/3 mol%, Eu_2_O_3_. Chandrasekhar et al. [50] and Ntwaeaborwa et al. [51] reported similar findings for europium-modified ZnO semiconductors. The presence of Eu^3+^ in the ZnO lattice can explain the variations in E_g_ values. The incorporation of Eu^3+^ ions as substitutes at Zn site introduces 4f states into the ZnO band gap [52]. These states are situated near the conduction band edge of ZnO and, unlike non-metal and transition metal ion dopants, do not hybridize with O2p states and Zn3d states due to the strong shielding effect of the 5s^2^ and 5p^6^ orbitals [53]. Nevertheless, spectroscopic data reveal a distinctive intra-shell luminescence transition of europium-modified, indicating that weak interactions with the environment cause the 4f states of europium to become somewhat delocalized upon substitution [54]. Because the influence of Zn3d states is lessened, the observed decrease in E_g_ value can be understood as a lowering of the conduction band minimum of ZnO. Therefore, the lowest E_g_ value at 3 mol% indicates that the dopant concentration has an optimal effect, most likely by raising the density of 4f states of Eu^3+^ replacing zinc ions in the ZnO lattice.

### 2.3. Tribocatalytic Decomposition of Paracetamol

**Figure 8:**
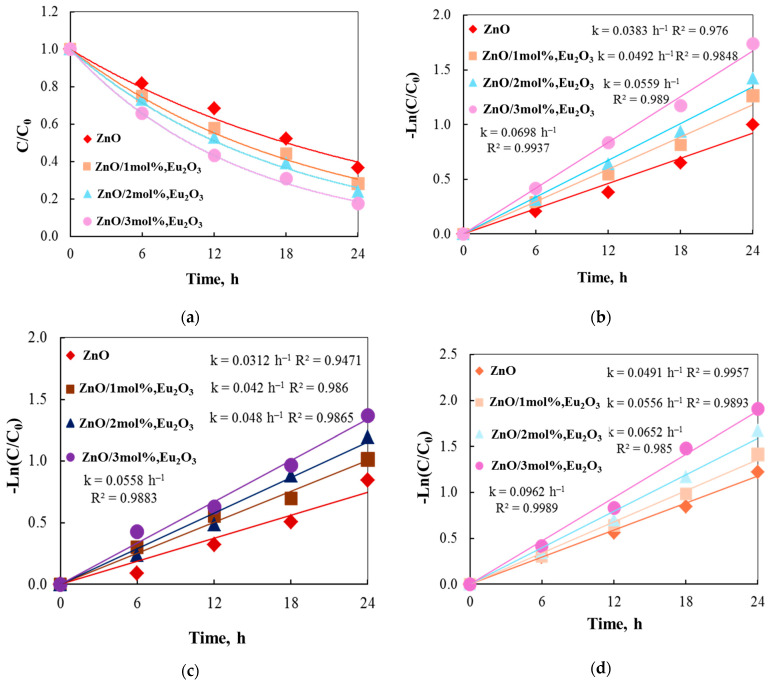
(**a**) Tribocatalytic degradation of paracetamol solution using ZnO and ZnO/Eu_2_O_3_ particles by magnetic stirring conditions at 300 rpm with one stirring rod; (**b**) kinetic fitting. The effect of various stirring speeds (**c**) 100 and (**d**) 500 rpm on the decomposition of paracetamol.

The tribocatalytic decomposition of paracetamol without light, utilizing a magnetic stirrer with one PTFE magnetic bar at 300 rpm, is employed to estimate the tribocatalytic efficiency of ZnO and ZnO/Eu_2_O_3_ particles. Each friction test uses the same drug concentration of 15 ppm. The concentration of the drug solution was determined using the PCA molecule’s distinctive absorbance at 243 nm. Figure 8a shows the tribocatalytic degradation of a paracetamol initially at a rotation speed of 300 rpm using pure and europium-modified ZnO particles. The results of the degradation process demonstrated the drug’s breakdown under the tribocatalytic action. After a 24 h friction period, the ZnO/3 mol% Eu_2_O_3_ particles degraded 82.44% of the paracetamol.

The catalytic efficiencies of ZnO/Eu_2_O_3_ particles are higher than those of pure ZnO. The findings indicate that tribocatalysis is significantly influenced by the morphological surface, crystallite size, and E_g_ values. The Ln(C_t_/C_0_) = −kt pseudo-first-order approximation yields the rate constant values (Figure 7b), which are consistent with the trend. The ZnO sample (k = 0.0383 h^−1^) has a lower reaction rate than the ZnO/1 mol% Eu_2_O_3_ sample (k = 0.0492 h^−1^). The catalyst with the highest molar concentration of europium has the highest rate constant (k = 0.0698 h^−1^).

To investigate the factors that influence the breakdown of paracetamol, a suspension of analgesic and ZnO and ZnO/Eu_2_O_3_ samples was agitated at different speeds and with varying numbers of stirring rods. The data are summarized in Figure 8 and Table 4. The logarithm of the concentration ratio with ZnO and ZnO/Eu_2_O_3_ particles is displayed in Figure 8 using a single Teflon stirring rod at varying stirring speeds. The rate constant and the decomposition of paracetamol using ZnO/3 mol% Eu_2_O_3_ at 500 rpm are k = 0.0962 h^−1^ and D = 85.1% after 24 h of stirring. At 300 and 100 rpm, the decomposition ratios are k = 0.0698 h^−1^ and D = 82.4%, k = 0.0558 h^−1^ and D = 74.5%, respectively. The data suggest that a higher stirring speed could produce more friction energy, which would be advantageous for the breakdown of drugs. Increases in rotation speed can enhance the contact probability between the drug molecules and the catalyst, promoting the tribocatalytic reaction and boosting the tribocatalytic efficiency. Similarly, when Dong et al. [55] studied the tribocatalytic degradation of RhB by Bi_2_WO_6_, they discovered that the degradation efficiency of dyes improved with an increase in rotation speed. However, if the rotation speed is too high, the stirrer may rebound during stirring and splash the catalyst onto the beaker wall, resulting in a decrease in catalytic efficiency. The aforementioned research has demonstrated not only the significance of magnetons in the tribocatalytic experiments but also a strong correlation between the generation of tribocatalytic charges and the frequency, friction area, and charge transfer efficiency between materials.

The decomposition results obtained with varying quantities and types of stirring rods at a 500 rpm stirring speed are displayed in Table 4. The decomposition ratio with one Teflon stirring rod is the lowest, while the decomposition ratio with three Teflon stirring rods reaches the maximum percentage of paracetamol degradation. With three Teflon stirring rods, there was a noticeable increase in the decomposition ratio, which can be attributed to the increased total contact area. Two 1-mm-thick PVC belt rings were wrapped around a stirring rod to confirm the impact of total contact area on the decomposition ratio. This alteration prevents the stirring rod from making contact with the suspension’s glass beaker bottom, as indicated in Table 4, which leads to a low decomposition ratio of about 25.8%. It offers proof that tribocatalytic drug breakdown is significantly influenced by the total contact area.

The contact area between the stirring rod and the catalyst, and the reactor bottom is increased by the greater number of stirring rods. Consequently, the tribocatalytic efficiency will rise as the number of stirring rods increases. Table 4 demonstrates that employing two stirring rods significantly enhances the tribocatalytic degradation efficiency of paracetamol compared to using only one stirring rod. The significance of friction between the stir bar and the vessel bottom is further illustrated by the three stirring rods.

The higher surface area, which provides more active sites for participation and permits greater adsorption of drugs in the reaction, may help to separate tribogenerated charge pairs and enhance carrier participation in the redox reaction, both of which may lead to improved tribocatalysis efficiency [56]. When ZnO absorbs mechanical energy during friction, excited e^−^ represents electrons, while the resulting h^+^ signifies holes. O_2_^−^ superoxide radicals are generated when oxygen molecules interact with electrons during the degradation of the drug. Following their interaction with OH^−^, the holes convert into hydroxyl radicals, or OH^•^. The enhanced activity of the europium-modified ZnO sample may be attributed to the efficient electron–hole separation across the ZnO/Eu_2_O_3_ interface and the increased generation of O_2_^−^ and OH^•^ radicals. The higher adsorption of hydroxyl ions onto the ZnO surface and the greater number of oxygen vacancies in the Eu-modified ZnO, resulting from the differing charge and electronegativity of europium and zinc ions, contribute to the higher efficiency [26]. The formation of OH^•^ is facilitated by the reaction between the holes and OH^−^. Organic contaminants on the surface of europium-modified ZnO are degraded by strong non-selective oxidants such as hydroxyl radicals and other tribogenerated active species [57,58]. Higher catalytic efficiency is achieved when ZnO is modified with the europium oxide phase, likely due to the suppression of tribogenerated charge recombination. The incorporation of the europium phase is advantageous as it traps electrons, inhibits electron-hole recombination reactions, and increases the quantity of superoxide and hydroxyl radicals, all of which accelerate the degradation of pollutants.

**Figure 9:**
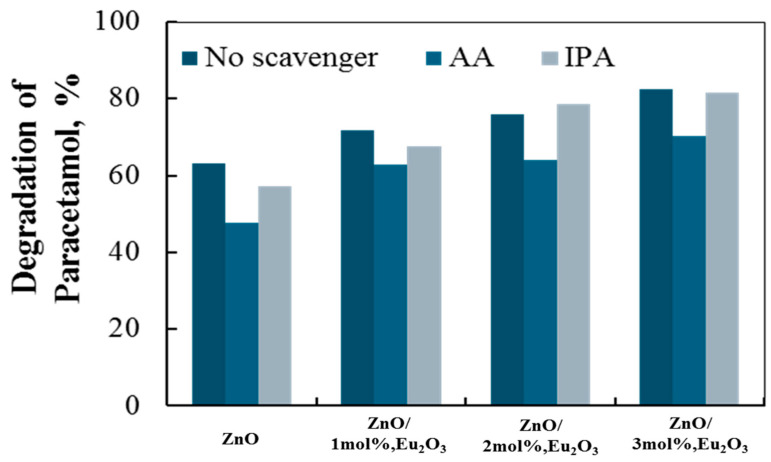
Scavengers’ effects on the decomposition of paracetamol in the tribocatalysis process using ZnO, ZnO/1 mol% Eu_2_O_3_, ZnO/2 mol Eu_2_O_3_, and ZnO/3 mol% Eu_2_O_3_ particles.

A radical scavenger assay that we conducted provides evidence for the role of the superoxide and hydroxyl radicals that are involved. Figure 9 displays the data. The addition of ascorbic acid (AA) and isopropyl alcohol (IPA) scavengers, which capture the corresponding reactive species, allowed for the quantification of the roles that superoxide and hydroxyl radicals play in the degradation of paracetamol [59,60].

Figure 9 shows that adding AA and IPA to the four tribocatalyst systems had comparable effects, with the former showing a more noticeable inhibition. This suggests that the superoxide radical has a greater impact on the paracetamol tribo-degradation rate.

**Figure 10:**
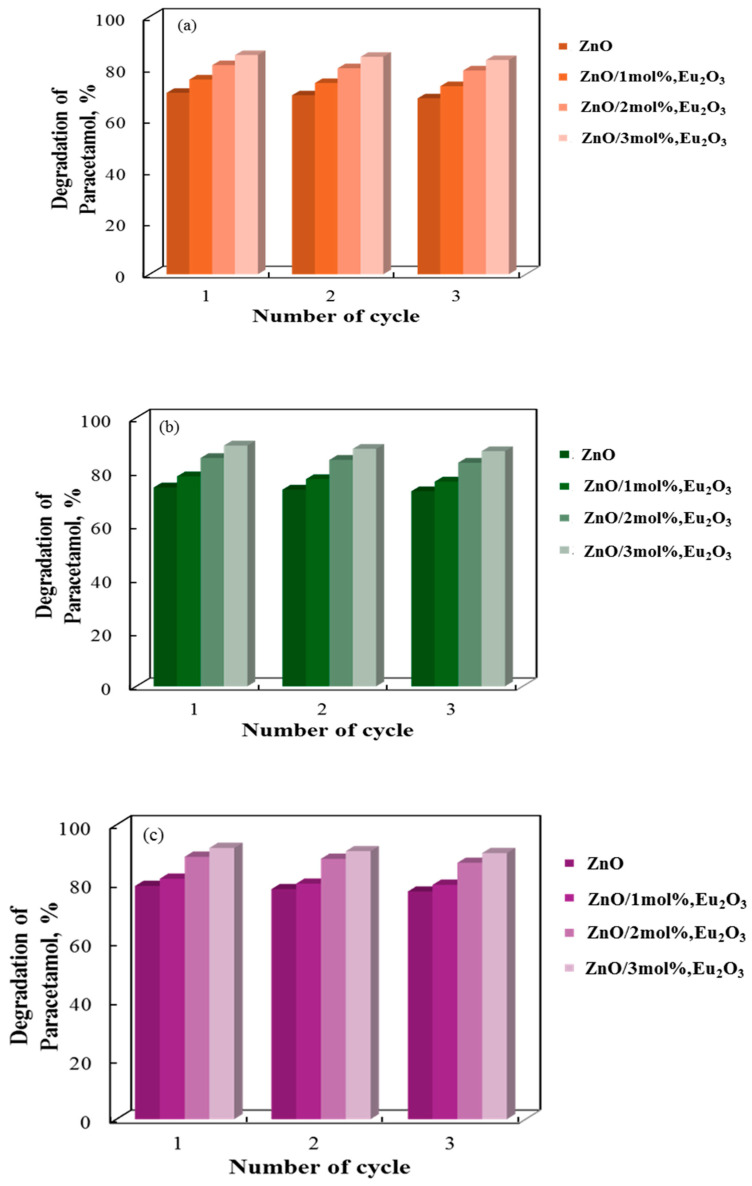
Decolorization rate of paracetamol at 500 rpm for three successive cycles using (**a**) 1 rod, (**b**) 2 rods, and (**c**) 3 rods.

A three-cycle investigation into the recyclability of tribocatalysts made of pure and europium-modified zinc oxide is depicted in Figure 10. Following three cycles in distilled water, the tribocatalytic decomposition of paracetamol decreased by approximately 2% for all catalyst types, indicating that the catalytic performance of the tribocatalysts diminished slightly with each cycle. Despite this decrease, the stability of the drug decomposition cycle of the sol-gel samples was found to be good. These results demonstrate their potential for repeated use in the breakdown of paracetamol. The most stable and effective catalyst over many cycles is Eu_2_O_3_, although ZnO/3 mol% shows a slight decrease with repeated use.

## 3. Materials and Methods

### 3.1. Chemicals

Zinc acetate dihydrate (>99.0%), 1-propanol (>99.0%), triethylamine (>99.5%), and europium oxide (>99.0%) were from Fluka (Buchs, Switzerland). Distilled water was used in all experiments. All other chemicals and reagents were of analytical reagent grade. Paracetamol (C_8_H_9_NO_2_, *λ*_max_ = 243 nm, Teva, Dupnitsa, Bulgaria) was chosen as the model pollutant for the tribocatalytic experiments due to its extensive application in the real world.

### 3.2. Synthesis of Sol-Gel ZnO and ZnO/Eu_2_O_3_ Particles

Four series of ZnO doped with Eu_2_O_3_ (0, 1, 2, and 3 mol%) powders were produced using a simple and eco-friendly sol-gel process. Zinc acetate dihydrate (0.5 g), ethylene glycol (0.15 mL), 1-propanol (20 mL), and triethylamine (0.35 mL) were mixed in a round-bottom flask with a reflux condenser and stirred (300 rpm) at room temperature for 15 min. The resulting solution was stirred for 60 min at 80 °C. ZnO tribocatalyst modified with Eu^3+^ (1 mol%) was prepared using the same method and conditions. Europium oxide was separately dissolved in 1-propanol and sonicated for 20 min to enhance dispersion. The pure reaction mixture was then added to this sonicated suspension of europium oxide, ensuring uniform doping. The resulting ZnO and ZnO/Eu_2_O_3_ particles were separated by centrifugation at 6000 rpm for 30 min, washed twice with pure 1-propanol, and dried in open air to yield powders. The remaining catalysts (ZnO/2 mol% Eu_2_O_3_ and ZnO/3 mol% Eu_2_O_3_) were prepared under the same optimal conditions.

### 3.3. Methods

A scanning electron microscope (SEM, JSM-5510, JEOL, Krefeld, Germany) was used to image the obtained samples in order to examine their morphology and microstructure. The samples were analyzed using energy-dispersive X-ray spectroscopy (EDX, detector: Quantax 200, Bruker Resolution 126 eV, Berlin, Germany) for elemental analysis or chemical characterization. Transmission Electron Microscopy (TEM) analyses were conducted on a JEOL JEM 2100 instrument (Akishima, Japan) at an accelerating voltage of 200 kV to analyze the morphology of the samples. The samples were prepared by dispersing them in ethanol for six minutes after ultrasonically grinding them. The suspensions were dripping onto standard carbon/Cu grids. The presence of all participating elements in the examined samples was further supported by elemental mapping studies using X-ray energy dispersive spectrometry (XEDS). Crystallinity and phase composition of the catalysts were analyzed using XRD (Siemens D500 with Cu Kα radiation, Karlsruhe, Germany). The average crystallite sizes were estimated using Scherrer’s equation. Rietveld analysis was executed employing PowderCell [61], and the March-Dollase texturing model [62] was used to examine whether the pure and Eu-modified ZnO samples showed signs of preferential orientation. Using a Zeiss Evo 15 microscope (Bruker Resolution 126 eV, Berlin, Germany), the energy dispersive X-ray spectroscopy (EDS) analysis was performed. X-ray photoelectron spectroscopy (XPS) was carried out using an ESCALAB MkII (VG Scientific, now Thermo Scientific, Manchester, UK) electron spectrometer at a base pressure in the analysis chamber of 5 × 10^−10^ mbar (during the measurement, 2 × 10^−9^ mbar), with an AlKαX-ray source having excitation energy hα = 1486.6. The pass energy of the hemispherical analyzer was 20 eV for O 1s and Zn2p spectra, whereas for Eu3d 50 eV pas energy of the analyzer was used because of the weak spectrum intensity resulting from the low concentration of europium on the surface. The instrumental resolution is about 1 eV for the photoelectron peak, as determined from the full width at half maximum (FWHM) of the Ag3d_5/2_. Data analysis was conducted using SpecsLab2 CasaXPS software (2.3.25PR1., Casa Software Ltd., Tokyo, Japan). The processing of the measured spectra includes a subtraction of X-ray satellites and Shirley-type background [61]. The peak positions and areas are evaluated by a symmetrical Gaussian-Lorentzian curve fitting. The relative concentrations of the different chemical species are determined based on the normalization of the peak areas to their photoionization cross-sections, calculated by Scofield [62,63]. EPR measurements were performed using a standard TE011 cylindrical resonator that came with a JEOL JES-FA 100 EPR spectrometer (Tokyo, Japan). Under the following conditions, the EPR spectra were recorded at room temperature: a modulation frequency of 100 kHz, a microwave power of 6 mW, a modulation magnitude of 0.2 mT, a time constant of 0.1 s, and a sweep time of 2 min. Raman spectra of the ZnO and ZnO/Eu samples were acquired using a ThunderOptics TO-ERS-532 system, equipped with a 20× microscope objective and a 532 nm laser source operating at 30 mW power. The spectra were collected with an integration time of 2000 ms (Thunder Optics S.A.S., Montpellier, France). Raman spectral data acquisition and processing were performed using Spectragryph software (version 1.2.16.1).

### 3.4. Tribocatalysis for Degradation of Paracetamol

Tribocatalysis was employed to break down a 50 mL solution of paracetamol. The drug was made in a 100 mL glass beaker with distilled water and a magnetic stirrer. Without light, the tribocatalytic reaction was conducted at a steady room temperature of 23 ± 2 °C. Initially, there were 15 ppm of analgesic present. Fifty mg of catalyst (pure or Eu^3+^-modified ZnO) was added to a glass reactor containing drug solution. Next, a magnetic bar sealed with polytetrafluoroethylene (PTFE) was used to stir the suspension magnetically. For 30 min in the dark, the resulting mixture was magnetically agitated with a PTFE to attain adsorption equilibrium between the tribocatalysts and the paracetamol solution. It then triggers the glass reactor, which initially spins at 300 revolutions per minute. Regularly, two-milliliter aliquots of the reaction solution were collected. After that, the tribocatalyst was centrifuged at 6000 rpm. This procedure was comparable to every other decomposition performance test, except for variations in the number of magnetic rods (2 and 3), magnetic stirring conditions (100 and 500 rpm), and catalyst types (ZnO/Eu (0, 1, 2, and 3 mol% powders). Friction between the catalyst and the vessel or between the stirrer and the reactor bottom may cause electron transfer during the stirring process. As a result, the vessel’s material is now considered during the tribocatalysis process, as the vessel significantly influences the tribocatalytic efficiency. Polytetrafluoroethylene (PTFE) has been found to be able to absorb more electrons during the friction process than glass and polypropylene (PP) [64]. As a result, the catalyst has higher tribocatalytic efficiency when it comes into contact with the PTFE beaker and PTFE stirrer, releasing more electrons. All these studies related to the type of reaction vessel will be examined in other future experiments of ours. Thermo Scientific’s Evolution 300 spectrophotometer (Madison, WI, USA) was used to acquire paracetamol’s UV–vis spectra in the 200–600 nm range.

A scavenger test was used to look into the reactive species that were causing the paracetamol to degrade. As scavengers, isopropyl alcohol (IPA) and ascorbic acid (AA) were employed to absorb hydroxyl and superoxide radicals, respectively. Six milligrams of each scavenger were used independently in order to pinpoint the precise reactive species that caused the organic drug (50 mL) to degrade due to tribocatalysis.

## 4. Conclusions

In conclusion, three different types of ZnO sol-gel particles modified with Eu_2_O_3_ (0, 1, 2, and 3 mol%) are employed to achieve strong tribocatalysis for the decomposition of paracetamol by harnessing the friction energy from stirring. Among these, the ZnO/3 mol% Eu_2_O_3_ composite demonstrated the most efficient degradation and the highest specific surface area. When the drug is stirred with three PTFE rods for 24 h at 500 rpm, the tribocatalytic drug decomposition ratio reaches approximately 92%. The influence of various factors, such as stirring speeds and the quantities and types of stirring rods, on the breakdown of paracetamol is assessed. The data show that electrons and holes in ZnO and ZnO/Eu_2_O_3_ particles are efficiently excited by mechanical energy absorbed during friction, resulting in effective drug decomposition. An environmentally friendly and promising method of using mechanical energy from the environment to combat pollution is the tribocatalytic effect. Tribocatalysis facilitates drug degradation, creating new opportunities for controlling environmental contamination.

## Figures and Tables

**Table 1:** The structural parameters calculated from XRD patterns of the ZnO and ZnO/Eu_2_O_3_ samples.

Tribocatalysts	ZnO Phase		Eu_2_O_3_ Phase	
Crystal. Size Parameters	Vol. Mi- % Crostrain	Crystal. Size Parameters	Vol. At. % %	Mi- Crostrain
**ZnO:** 42.4 a, b: 3.2407 c: 5.2017	100 1.8 × 10^−3^	―	―	―
**ZnO/1 mol% Eu_2_O_3_** 39.0 a, b: 3.2484 c: 5.2043 **ZnO/2 mol% Eu_2_O_3_** 36.0 a, b: 3.2475 c: 5.2032 **ZnO/3 mol% Eu_2_O_3_** 30.2 a, b: 3.2485 c: 5.2039	97.8 3.3 × 10^−4^ 94.8 2.1 × 10^−4^ 93.7 9.2 × 10^−4^	33.4 a, b, c: 10.8485 34.1 a, b, c: 10.8592 26.8 a, b, c: 10.8587	2.2 0.68 5.2 1.65 6.3 2.01	1.5 × 10^−3^ 9.9 × 10^−4^ 2.5 × 10^−1^

**Table 2:** Surface atomic concentrations (at.%), binding energies (eV), and corresponding oxidation states/bond types of elements in xEu/ZnO samples (x = 0, 1, 2, and 3 mol%) prior to tribocatalytic testing.

Before O 1s Tribocatalysts	Zn2p		Eu3d_5/2_	
BE, eV	Conc., BE, at% eV	Conc., at%	BE, eV	Conc., at%
ZnO 530.6 531.6 532.5	27.67 1021.7 15.78 6.76	49.80	―	0.00
ZnO/1 mol% Eu_2_O_3_ 530.7 531.6 532.5 ZnO/2 mol% Eu_2_O_3_ 530.7 531.7 532.7 ZnO/3 mol% Eu_2_O_3_ 530.7 531.5 532.4	29.40 1021.7 14.27 6.21 28.88 1021.7 14.75 5.79 28.32 1021.7 15.30 8.32	50.16 50.35 42.73	~1135.0 ~1135.9 ~1136.0	0.17 0.23 0.32

**Table 3:** Surface atomic concentrations (at.%), binding energies (eV), and corresponding oxidation states/bond types of elements in ZnO/xEu samples (x = 0, 1, 2, and 3 mol%) after tribocatalytic testing.

After O 1s Tribocatalysts	Zn2p		Eu3d_5/2_	
BE, eV	Conc., BE, at% eV	Conc., at%	BE, eV	Conc., at%
ZnO 530.7 531.6 533.0	24.46 1021.7 19.19 7.28	49.06	―	0.00
ZnO/1 mol% Eu_2_O_3_ 530.7 531.7 532.7 ZnO/2 mol% Eu_2_O_3_ 530.7 531.7 532.9 ZnO/3 mol% Eu_2_O_3_ 530.7 531.6 532.9	28.70 1021.7 16.12 7.07 31.76 1021.7 13.59 8.30 28.05 1021.7 19.00 6.70	47.89 46.08 46.09	~1135.6 ~1135.4 ~1134.5	0.21 0.27 0.16

**Table 4:** The values of rate constants and percentages of paracetamol degradation at 500 rpm using various amounts and types of PTFE rods.

Tribocatalysts 1 Rod	2 Rods		3 Rods	
k, h^−1^	D, % k, h^−1^	D, %	k, h^−1^	D, %
ZnO 0.0383	70.45 0.0564	74.07	0.0671	74.07
ZnO/1 mol%, Eu_2_O_3_ 0.0492 ZnO/2 mol%, Eu_2_O_3_ 0.0559 ZnO/3 mol%, Eu_2_O_3_ 0.0698	75.62 0.0647 81.20 0.0795 85.12 0.0939	78.20 85.02 89.67	0.0778 0.0969 0.1054	81.71 89.15 92.15

## Data Availability

The original contributions presented in this study are included in the article. Further inquiries can be directed to the corresponding author(s).
